# The effect of bereavement groups on grief, anxiety, and depression - a controlled, prospective intervention study

**DOI:** 10.1186/s12904-016-0129-0

**Published:** 2016-07-12

**Authors:** Ulla Näppä, Ann-Britt Lundgren, Bertil Axelsson

**Affiliations:** Department of Nursing Sciences, Mid Sweden University, 831 25 Östersund, Sweden; Centres of Surgery, Östersund Hospital, Östersund, Sweden; Centre of Patient Safety, Östersund Hospital, Östersund, Sweden; Department of Radiation Sciences, Unit of Surgery - Östersund, Umeå University, Umeå, Sweden

**Keywords:** Bereavement, Cancer, Family caregivers, Oncology, Palliative care, Support

## Abstract

**Background:**

Bereavement groups are believed to be beneficial as preventive interventions to reduce the development of complicated grief for people at risk after the death of a significant other. This study aimed to investigate whether measurable effects on grief, anxiety, and depression could be detected in those participating in bereavement groups compared to non-participating controls.

**Methods:**

Questionnaires covering the Texas Revised Inventory of Grief (TRIG), the Hospital Anxiety and Depression Scale (HADS), and background questions were handed out pre-intervention, five weeks and one year post-intervention to bereaved caregivers invited to bereavement groups. The results were analysed with non-parametric methods.

**Results:**

A total of 124 individuals answered the questionnaires, and were divided into three categories: participants, non-participants unable to participate, and non-participants not wanting to participate in bereavement groups. At the one-year follow up, participants and those unable to participate reported higher levels of grief and were more anxious than those not wanting to participate. Depression did not differ between the groups.

**Conclusions:**

Participation in bereavement groups did not produce any effects on grief, anxiety, or depression in comparison to non-participants who were unable to participate. Non-participants who did not want to participate reported lower levels of grief and anxiety than the other two groups.

## Background

The World Health Organization’s definition of palliative care underlines the need for a support system to help the family cope during the patient’s illness and in their bereavement [1]. In its broadest sense, “family” includes both family members and others who have a caring bond with the dying person, and hence “significant other” is probably a better term. Most significant other are extensively emotionally involved throughout the disease course, and in some cases are also involved in care provision [2–4]. The loss of a close person, independent of the reason, is usually a traumatic experience. All people grieve differently; some need professional help while others are resilient in their loss and do not require special interventions [5–7]. It has been debated if grief should be medicalised or not. Dyregrov [8] means that grief should not be overgeneralised; efforts should be put in “separating sad, but ordinary, experiences from those that often lead to serious clinical illness (p. 9)” [8].

Grief is defined as an internal experience in reaction to the loss of something loved and valued [9]. This loss can lead to the experience of losing control, and experiencing overwhelming emotions such as, fear, loneliness, loss of identity, and other difficult feelings [10]. However, despite this misery, most grieving individuals do not develop mental and or physical complications [7]. A minor number of significant other in the bereavement period show increased risk for hospitalisation and death as well as depression, mental illness, and substance abuse [11]. Ideas and assumptions about grief and coping with grief have changed over time. Lindemann stated in the 1940s that if normal grief is shared with professional help, it is possible to settle an uncomplicated grief reaction in four to six weeks [5]. More recently, Reynolds et al. argued that grief and depression are normal emotions in bereavement, but that symptoms of grief resolve more slowly than medically treated depression [12]. For a minority of people, when normal grief turns into complicated grief the result may be considerable functional impairment, co-morbid depression, and/or other anxiety disorders [13]. Lindqvist and Tishelman [14] suggests that patient and public involvement is needed to inspire a salutogenetic approach in palliative care, including bereavement [14]. For many years it has been debated whether providing support for bereaved informal caregivers should be recommended in palliative guidelines [1, 15, 16]. The methods and effects of bereavement support have, however, been debated. In hostile and dysfunctional families, bereavement support may even worsen grief and depression [17]. Another question in clinical practice is whether those who may benefit from bereavement support the most are among those who do not participate in the bereavement support activities that are offered to them.

Bereavement groups are believed to be beneficial as preventive interventions from social and economic standpoints. Participation is likely to be more acceptable and less threatening to potential recipients than professional interventions linked to psychiatry. Costs can be low, since groups are usually led by staff or volunteers rather than mental health professionals [18]. Group interventions may also lessen the likelihood of development of complicated grief including risks of mental health problems such as major depression or anxiety [19], as well as sudden death, suicide, lack of social support, and/or isolation [20, 21].

The emotional stress and grieving experienced by informal caregivers after the patient’s death is well known, and the recommended practice of bereavement support seems intuitively sound [22]. Nevertheless, the evidence base for measurable effects of this intervention needs to be complemented by more prospective controlled intervention studies using validated and well-known questionnaires. The primary aim of this study was to investigate whether we could detect measurable effects on grief, anxiety, and depression in those participating in bereavement groups in comparison to non-participating controls. A secondary aim was to use the same methodology to compare non-participants who had wanted to participate but could not for practical reasons with non-participants who did not want to participate.

## Methods

The study setting was a county in northern Sweden with 126 500 inhabitants (<1 % of the full Swedish population) and an area of 49 443 km^2^ (2.6 inhabitants per km^2^), consisting of mountains, rural areas, and one urban area close to the only city in the middle of the county. The county is served by one hospital and a specialised palliative home care team based at the hospital that treats approximately 150 patients per year. Consecutive invitations were sent 3–6 months after death of all palliative patients to one significant other per patient offering participation in a bereavement group. During the two-year period of this study, invitations resulted in ten bereavement groups being held at the hospital, each with three to eleven participants and jointly led by a nurse from the palliative care unit, a social worker, and a clergy member or deacon from the hospital church. All facilitators were salaried entirely from tax money, without any charity contributions. Social workers, clergy members, and deacons are formally trained for counselling during their education, while registered nurses touch on the subject during their basic training. The professionals tutoring the bereavement groups were either members of the palliative care unit (nurses) or members of the hospital staff (social workers, clergy members, deacons), and all had several years of clinical experience of counselling family members and significant other to palliative patients. Before the start of each bereavement group, the relevant facilitators met to discuss aspects of the forthcoming group including the methodology, the facilitating approach, and the main theme of the planned meeting. The bereavement group themes and methodology followed the rationale recommended by the hospital churches in Sweden [23]. The underlying theory was the potentially positive effect of verbalisation and re-exposure of the grief experience in a safe group context [23–26]. Each bereavement group met once a week for five weeks; meetings included afternoon tea and lasted for two hours. Each of the five meetings had a predefined theme: 1) presentation of the methodology and introduction of the members in the group, 2) the time of the illness until death, 3) the time of death, 4) the time after death and the funeral, and 5) depicting a metaphorical picture of the deceased and the significant other’s life together. The meetings were held in a separate room with participants and facilitators seated around the same table. The role of the facilitators was not to lecture, but to listen, let the participants discuss, and make sure everyone had a chance to express their thoughts and emotions.

Two weeks after being invited to the bereavement group by the facilitators, each invited person was sent a package including an informed consent form, a questionnaire containing background questions, the Texas Revised Inventory of Grief (TRIG) questionnaire, and the Hospital Anxiety and Depression Scale (HADS) questionnaire; these questionnaires were intended to be completed before the start of the bereavement groups. In the information letter, all invited persons were also informed that new packages of questionnaires would be sent on two more occasions unless they declined the informed consent.

**Fig. 1:**
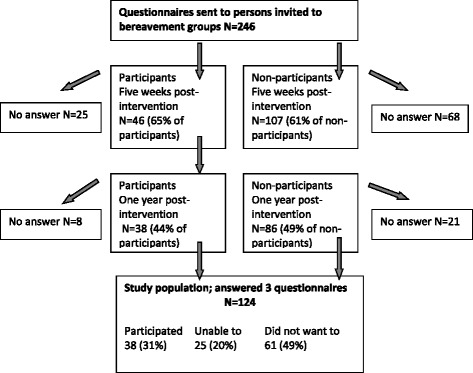
Participation in bereavement groups and answers to questionnaires

Based on earlier experiences, we were well aware that only a small number of those invited would participate in the bereavement groups offered. However, this also gave an opportunity to investigate any differences between participants and non-participants. Five weeks after the last meeting of the bereavement group, both participants and non-participants were sent a post-intervention package including the TRIG and HADS questionnaires. The final follow-up package including TRIG and HADS was sent about 1 year after the intervention to persons who had answered both earlier questionnaires (Fig. 1). At all occasions, one reminder was sent to those who had not answered within two weeks. The inclusion criterion for this study was completed questionnaires at all three occasions.

Socio-demographic questions were constructed by the authors, and covered gender, age, education, working status, relation to the deceased, time since death, living alone or not, participation in bereavement groups, and desire to participate. Open questions provided the respondents with an opportunity to comment on their experiences of any effects of participation in the bereavement groups. For non-participants, these questions gave the opportunity to describe other kinds of grief experiences and support.

The TRIG questionnaire is a 21-item patient-completed questionnaire that measures an individual’s experience of grief. It consists of two scales: Past Behaviour and Present Feelings. In this study, only the Swedish version of the Present Feelings scale was used. Faschingbauer et al.’s [27] psychometric tests of the instrument has revealed that thoughts, feelings, memories, opinions and attitudes are all being tapped by this second part of the instrument. Since the main focus in this study was to assess the bereaved person’s present grief reactions in a longitudinal design, only the second part of the instrument was used. Thirteen items were assessed on a Likert scale with five response alternatives: “completely true”, “mostly true”, “true and false”, “mostly false”, and “completely false”. TRIG measures grief from several different angles, including thoughts, feelings, memories, opinions, and attitude. It is used to identify grief reactions, and is suitable for measuring grief over time. It is known to show gender differences, and to reflect that spouses are likely to experience greater levels of grief than other relatives. TRIG presents grief as either normal or severe. The lower score, the more severe grief. Scores up to 39, i.e. the 50th percentile, are defined as severe grief [27]. In this study, grief was defined as either normal or severe according to Faschingbauer et al., who stated that a person with severe grief suffers from a greater life disruption [27].

To detect anxiety and depressive mood, the Swedish version of the HADS was used. The HADS consists of 14 items measuring levels of anxiety and depression in two separate subscales. Each item has four response categories, reflecting a continuum of increasing level of emotional distress in the somatically ill. Total scale scores range from 0 (no symptoms) to 21 (maximum distress) for both depressive mood and anxiety. This patient-completed scale can reliably detect the presence of depressive mood and anxiety. Its subscales have been shown to be valid measures of the severity of emotional distress, with 0–7 points indicating “non-cases”, 8–10 points indicating “doubtful cases”, and >10 points indicating “cases” [28, 29].

Numerical data were analysed using version 22 of the SPSS software package (IBM Corporation). Pearson’s chi-square test and Fisher’s exact test were used to detect differences between groups, and Wilcoxon’s signed rank test was used to detect longitudinal changes. Ranges, means, and 95 % confidence intervals were calculated. Groups were defined as “participant in bereavement group”, “non-participant unable to participate”, and “non-participant not wanting to participate”. Missing answers were considered as missing data, and not included in the analysis.

## Results

Questionnaires were sent to 246 invited persons (155 [63 %] women and 91 [37 %] men) at pre-intervention and at post-intervention five weeks after the end of the bereavement group, and to 153 persons (101 [66 %] women and 52 [34 %] men) at follow-up one year after the intervention. Questionnaires were completed at all three occasions by 124 persons, who formed the population of this study: 84 (68 %) women and 50 (32 %) men (Fig. 1). The only information available about the persons not included in the study was gender and distance from the hospital where the bereavement groups were held. No differences were found in either of these factors compared to the study population (*N* = 246; gender *p =* .116; distance *p* = .550).

Persons willing to attend bereavement groups over a two-year period formed 10 groups, all of which held meetings as planned. A total of 62 persons participated in the groups (3–11 participants per group, median 7 participants). A full set of three questionnaires was received from 38 persons who participated in the bereavement groups (61 % of all participants) as well as 86 persons who had not participated in the bereavement groups, of whom 25 (29 %) were unable to participate and 61 (71 %) did not want to participate. Socio-demographic data for the study population is shown in Table 1. Average attendance rate at the meetings was 83 %.

**Table 1:** Socio-demographic data for individuals who answered questionnaires at all three occasions (*n*=124)

	Participants *n*=38 (%)	Non-participants *n*=86 (%)	*p*
Gender			.175
Male	9 (24)	31 (36)	
Female	29 (76)	55 (64)	
Age (range 39–86)	Median 64.5	Median 63.5	.037
≤60	10 (26.5)	36 (42)	
61–70	18 (47)	21 (24)	
>70	10 (26.5)	29 (34)	
Relation to the deceased			.100
Spouse	28 (74)	56 (65)	
Child of the deceased	3 (8)	22 (26)	
Parent of the deceased	5 (13)	6 (7)	
Friend	2 (5)	1 (1)	
Other	0	1 (1)	
Distance to hospital	Mean <60 km	Mean 60–109 km	.026
<60 km	27 (71)	43 (50)	
60–109 km	9 (24)	22 (26)	
>109 km	2 (5)	21 (24)	
Working status			.029
Full time	6 (17)	27 (32)	
Part time	3 (8)	8 (9)	
Sick leave	5 (14)	1 (1)	
Retired	20 (55)	43 (50)	
Other	2 (6)	7 (8)	
Missing (2 persons)			
Highest education			.791
Primary school	9 (24)	27 (32)	
Secondary school	6 (16)	9 (10)	
University	8 (21)	19 (22)	
Vocational training	12 (31)	22 (26)	
Other	3 (8)	9 (10)	
Living			.162
Alone	29 (76)	54 (64)	
Not alone	9 (24)	31 (36)	
Missing (1 person)			

Among those included in the study, the groups of non-participants were more likely to be aged over 60 years (*p* = .037), lived further away from the city where the bereavement groups were held (*p* = .026), and were more likely to work full time (*p* = .029), compared to the bereavement group participants. No differences were found between participants and non-participants in terms of gender, relation to the deceased (spouse, child, parent, or friend), and grade of education. Participants were slightly more living alone, although the difference was not significant.

Non-participants (*n* = 86) were divided in two groups: *unable to participate* (*n* = 25) and *did not want to participate* (*n* = 61).

The most frequent reasons for persons unable to participate were overly-long distance and work (Table 2). The only difference in socio-demographic parameters between non-participants was that more persons younger than 61 years did not want to participate (*p* = .008).

**Table 2:** Reasons not to participate in bereavement groups

Reasons	Unable to participate *n*=25 (%)	Did not want to participate *n*=61 (%)	Total *n*=86 (%)
No answer	4 (16)	21 (34)	25 (29)
Overly-long distance	10 (40)	7 (12)	17 (20)
No need	0	13 (21)	13 (15)
Work	6 (24)	6 (10)	12 (14)
Health	2 (8)	4 (7)	6 (7)
Other support	0	6 (10)	6 (7)
Other reasons	2 (8)	1 (1.5)	3 (4)
Not at home	1 (4)	1 (1.5)	2 (2)
Do not want reminders	0	2 (3)	2 (2)

### TRIG scores in the different groups

A majority of the participants in this study (*n* = 91; 81 %) reported TRIG scores representing severe grief before the start of the intervention, regardless of future participation in bereavement groups (Table 3). At five weeks post-intervention, no differences could be seen in grief levels between participants and all non-participants. However, there was a difference between non-participants; persons who did not want to participate reported lower levels of grief than persons who were unable to participate (*p* = .004). At the one-year follow-up, participants and non-participants unable to participate still presented higher levels of grief compared to persons who did not want to participate (*p* = .039 and *p* = .018 respectively).

**Table 3:** TRIG grief scores for participants, persons unable to participate, and persons not wanting to participate in bereavement groups

	Participants *n* = 38 (%)		Unable to participate *n* = 25 (%)		Did not want to participate *n* = 61 (%)	
Grief score	Severe	Normal	Severe	Normal	Severe	Normal
Pre-intervention *n* = 112	28 (87)	4 (13)	20 (87)	3 (13)	43 (75)	14 (25)
range	13–35	40–51	16–38	43–61	15–39	44–61
-Mean TRIG	24.9	44.0	27.2	49.3	29.4	48.8
-Confidence interval (95 %)	22.2–27.6	36.1–51.9	24.5–30.0	24.2–74.5	27.3–31.4	31.4–51.3
Five weeks post-intervention *n* = 120	31 (82)	7 (18)	23 (96)	1 (4)	38 (66)	20 (34)
range	13–38	40–54	13–39	40	15–39	40–57
-Mean TRIG	25.5	44.4	26.6	40.0	29.0	46.7
-Confidence interval (95 %)	23.1–28.0	40.2–48.6	23.2–30.1		26.6–31.4	44.4–49.0
One year post-intervention *n* = 119	28 (78)	8 (22)	21 (84)	4 (16)	33 (57)	25 (43)
range	15–39	40–58	13–38	40–50	13–38	41–58
-Mean TRIG	27.8	45.9	27.2	43.5	28.5	47.6
-Confidence interval (95 %)	25.2–30.4	40.7–51.1	23.4–31.2	36.4–50. 6	26.3–30.7	45. 6–49.6

*p*-values are presented in the text

The proportion experiencing severe grief decreased in the whole study population between the pre-study assessment and the one-year follow-up (*p* = .002). However, only persons who did not want to participate had more pronounced grief reduction at five weeks (*p* = .014) and at one year (*p* = .002). No changes in TRIG levels were seen over time for participants and those unable to participate.

### HADS – anxiety scores in the different groups

In terms of anxiety, prior to the intervention 83 persons (69 %) were non-cases, 16 (13 %) were doubtful cases, and 21 (17 %) were cases (Table 4). Pre-intervention, participants experienced more anxiety than non-participants (*p* = .009). Five weeks post-intervention, participants (*p* = .009) and those unable to participate (*p* = .002) were more anxious than those who did not want to participate. At the one-year follow-up, participants did not differ from those who did not want to participate. However, those who were unable to participate were more anxious than those who did not want to participate (*p* = .006). Longitudinal levels of anxiety did not change at any time in any group. Most respondents reporting high levels of anxiety were seen among those who were unable to participate.

**Table 4:** HADS anxiety (HADA) and depression (HADD) scores for participants, persons unable to participate, and persons not wanting to participate in bereavement groups

	Participants *n*=38 (%)			Unable to participate *n*=25 (%)			Did not want to participate *n*=61 (%)		
Anxiety score	non cases	doubtful cases	cases	non cases	doubtful cases	cases	non cases	doubtful cases	cases
*Pre-intervention n=120*	20 (55)	10 (28)	6 (17)	12 (50)	2 (8)	10 (42)	51 (85)	4 (7)	5 (8)
range	0–7	8–10	12–20	0–7	8	11–18	0–7	8–10	11–17
- Mean HADA	4.2	8.8	14.5	3.7	8.0	14.4	3.5	9.0	13.2
- Confidence interval (95%)	3.2–5.2	8.2–9.4	11.4–17.6	2.2–5.1		12.5–16.3	2.8–4.2	7.7–10.3	10.1–16.3
*Five weeks post-intervention n=121*	23 (61)	5 (13)	10 (26)	12 (48)	6 (24)	7 (28)	49 (85)	6 (10)	3 (5)
range	0–7	8–9	11–20	1–7	8–10	13–18	0–7	8–10	11–15
- Mean HADA	3.5	8.2	14.3	4.2	9.0	14.7	2.8	9.2	13.3
- Confidence interval (95%)	2.6–4.4	7.6–8.8	12.4–16.2	3.0–5.5	8.1–9.9	12.9–16.5	2.2–3.4	8.4–10.0	8.2–18.5
*One year post-intervention n=124*	26 (68)	5 (13)	7 (18)	13 (52)	4 (16)	8 (32)	49 (80)	8 (13)	4 (7)
range	0–7	8–10	11–17	0–7	8–10	11–18	0–7	8–10	11–13
- Mean HADA	3.3	8.6	13.6	3.2	9.0	13.8	2.9	8.6	11.8
- Confidence interval (95%)	2.3–4.2	7.5–9.7	11.6–15. 6	1.8–4.6	7.2–10.8	11.4–16.1	2.2–3.5	8.0–9.2	10.2–13.3
Depression score	non cases	doubtful cases	cases	non cases	doubtful cases	cases	non cases	doubtful cases	cases
*Pre-intervention n=120*	25 (69)	5 (14)	6 (17)	15 (62)	5 (21)	4 (17)	47 (79)	11 (18)	2 (3)
range	1–7	8–10	11–19	0–7	8	12–18	0–7	8–10	11–18
- Mean HADD	4.0	9.0	14.0	4.2	8.0	13.5	3.5	9.3	14.5
- Confidence interval (95%)	3.2–4.8	8.1–9.9	11.1–16.9	2.8–5.6		8.7–18.3	2.8–4.2	8.8–9.7	N/A
*Five weeks post-intervention n=121*	27 (71)	5 (13)	6 (16)	16 (64)	3 (12)	6 (24)	46 (79)	6 (10)	6 (10)
range	0–7	8–10	11–19	0–7	8–10	11–16	0–7	8–9	11–19
- Mean HADD	3.8	8.4	14.0	3.2	8.7	12.5	3.1	8.2	14.0
- Confidence interval (95%)	2.9–4.8	7.3–9.5	10.6–17.4	2.0–4.5	5.8–11.5	10.5–14.5	2.4–3.7	7.7–8.6	10.6–17.4
*One year post-intervention n=124*	28 (74)	6 (16)	4 (10)	18 (72)	4 (16)	3 (12)	49 (80)	6 (10)	6 (10)
range	0–7	8–10	11–17	1–7	8–9	15–19	0–7	8–10	11–15
- Mean HADD	2.7	8.7	13.5	3.3	8.5	17.3	3.1	9.2	12. 7
- Confidence interval (95%)	1.9–3.5	7.8–9.5	9.3–17.7	2.1–4.5	7.6–9.4	12.2–22.5	2.4–3.8	8.4–10.0	11.0–14.4

*p*-values are presented in the text

### HADS – depression scores in the different groups

In terms of depression, prior to the intervention, 87 (70 %) individuals were non-cases, 21 (17 %) were doubtful cases, and 12 (10 %) were cases. No differences in levels of depression were detected between any of the groups either before or after the intervention (Table 3). Longitudinally, a trend of decreased levels of depression was seen from five weeks post-intervention to one year post-intervention among those unable to participate (*p =* .059). No other changes over time were detected.

The open question about the role the bereavement group played in the participants’ bereavement was answered by 33 of 38 persons (87 %). Most of the comments were positive, mentioning feelings of joint experiences and increased understanding of others’ reactions; they were not alone, or they were grieving in similar ways. Some comments gave advice to the leaders, and a few were negative. A comment by one of the participants summarises the concluding theme of the positive responses: *“The bereavement group meant a lot – talking – crying – breaking down – looking forward. It felt so nice and peaceful.”*

## Discussion

This study showed that most people are affected with severe grief in connection with the loss of a significant other, but also that this grief decreases significantly during the first year after the loss, confirming earlier studies [5, 22, 27, 30]. However, no measurable effects on grief, anxiety, or depression could be detected following participation in bereavement groups up to one year post-intervention. Accordingly, the results raise the question of whether grievers can actually benefit from bereavement groups with this specific methodology. One must keep in mind there are very different ways of grieving. Some persons may prefer retreat, or going on with their “business as usual”, while others may just benefit from exchange and sharing their experiences in a group. However, according to answers to the open questions, the bereavement groups seemed to produce positive effects which could not be captured by the chosen outcome measures, such as a deeper insight into the grieving process and a feeling of joint experience in grief. It cannot be told how anxiety, depressive mood and grief would have evolved in participants without the intervention.

Nearly half (49 %) of the individuals in our study stated that they had no need for bereavement group participation. They seemed to be able to handle their grief themselves and/or with support from their social network. The results of this study indicate that we as health care staff do not have to worry about most of those persons who decline participation in bereavement groups, as they show less severe grief and anxiety than others. Support from professional staff has been shown to be necessary only when the family network is dysfunctional, with poor communication [17, 30]. However, self-reported reasons of not wanting to participate may be a potential limitation of results. For example, depending on the level of psychological strain, one might regard geographical distance or lack of time as unaffordable obstacles to participation.

This study showed differences among non-participants, between those who were unable to participate and those who did not want to participate. The latter group experienced less grief and anxiety both at five weeks and one year post-intervention. It is notable that those unable to participate also had a tendency to experience more anxiety than the participants before the time of the intervention. One potential bias is that it is not known in this study to what extent significant others were parts of the caregiving for their dying loved one. Research shows that taking part in the caregiving gives a meaning of death and capacities for resilience [31, 32].

Bereavement group interventions are widely used by the Swedish Church, but the present study questions whether this concept is the best way to cater for people with severe grief. Other strategies could perhaps be a better way of providing support, and a more flexible offer regarding time of day and method of support. Use of, for example, the Internet, telephone calls, and personal meetings could possibly increase the likelihood of finding ways to help people who want to participate but are unable to for any reason. The major obstacles to participation in bereavement groups for those unable to participate were overly-long travel distance (40 %) and work (24 %).

Research shows that optimal bereavement support begins with significant others being a part of the dying process, and recognised as members of the health care team, regardless of their roles of responsibility and assistance in the care [2, 33]. Empirical evidence shows that early intervention provided to a distressed family before the death of a terminally ill patient can ease their bereavement and reduce depressive symptoms [9, 33, 34]. A study of a support group programme for relatives during the late palliative phase revealed a sense of belonging, created by sharing similar experiences and not being alone [35]. An open and honest discussion between caregivers, patients, and significant others about the impending death may improve the prospect of identifying probable cases of complicated grief, anxiety, and depression. To ensure that professional support is given to those who need it most, and with optimal effects, our suggestion would be that the bereavement support begins as soon as the individual at risk is identified; that is, ideally when the patient is still alive.

A limitation of this study is that we lack information about grief, anxiety, and depression among those 50 % who declined study participation. Reminding letters were sent once, but as stated earlier, these people were in a potentially vulnerable life situation which understandably may have decreased their willingness to participate in a scientific study. Respecting the choice of a person who has declined study participation is in accordance with the essence of the Helsinki declaration of Good Clinical Practice [36]. With regard to this, a 50 % response rate on three extensive packs of questionnaires during a period of one year must be seen as an acceptable answering frequency.

Another potential limitation of the study is that only 38 study participants received the intervention, which may be regarded as too low power to enable detection of an existing difference. Our clinical opinion is that if relevant differences in treatment effects are not detected in an intervention group of 10 to 15 persons, in spite of 10 hours counselling with three experienced facilitators, treatments are difficult to motivate as “value for money”. As this study compared 38 intervention persons with 86 controls, we regard the results as clinically interesting. However, attendance rate was high at meetings so the results may give a reasonable picture.

Usually, the respondents are those most in favour of the intervention; but despite this, the present study did not succeed in detecting an impact of the intervention. TRIG may seem to be a blunt assessment tool, only measuring “grief” or “severe grief” and not able to identify possible risk groups [37]. On the other hand, the HADS has been shown to be a valid scale measuring anxiety and depression separately, as originally suggested by its authors [27, 33]. Still, both questionnaires are used worldwide, allowing valid comparisons to be made with other studies. The differing findings from the open-ended question and the results from TRIG and HADS suggest that further qualitative research is needed.

## Conclusions

Before the bereavement group intervention, most respondents (81 %) reported levels of severe grief. Participants did not fare significantly better than non-participants. Those who did not want to participate reported less grief and anxiety than both participants and non-participants who had wanted to participate. No differences in levels of depression were detected between any groups either before or after the intervention.

Open-ended questions revealed anonymous, mostly positive effects of participation in bereavement groups which were not captured by the questionnaires measuring grief, anxiety, and depression. This underlines the need for a qualitative approach to gain better understanding and more in-depth insights of the subjective benefits experienced from participation.

## Abbreviations

HADS, the Hospital Anxiety and Depression Scale; TRIG, the Texas Revised Inventory of Grief
